# The dynamics of loneliness: A longitudinal exploration of transitions in Japan across the COVID-19 period, 2020–2024

**DOI:** 10.1371/journal.pone.0358269

**Published:** 2026-09-11

**Authors:** Jargalmaa Amarsanaa, Trinh Xuan Thi Nguyen, Mostafa Saidur Rahim Khan, Yoshihiko Kadoya

**Affiliations:** School of Economics, Hiroshima University, Kagamiyama, Higashihiroshima, Japan; UFMS: Universidade Federal de Mato Grosso do Sul, BRAZIL

## Abstract

**Background:**

Concerns about increased loneliness during the COVID-19 pandemic have been widely reported. However, long-term trajectories of loneliness remain insufficiently described.

**Objective:**

To examine annual trends and five-year transition patterns of loneliness in Japan from 2020 to 2024 using data from a nationwide online panel.

**Methods:**

We analyzed data from 1,673 adults who participated in all five annual survey waves from 2020 to 2024 and had complete information on the variables used in the analysis. Loneliness was measured using the three-item UCLA Loneliness Scale. Descriptive statistics were used to estimate annual prevalence and five-year transition patterns. Sensitivity analyses were conducted using a stricter loneliness threshold.

**Results:**

The prevalence of loneliness peaked in 2021, declined in 2022, and remained relatively stable from 2022 to 2024. Most respondents demonstrated stable trajectories over five years, either consistently reporting loneliness or consistently not reporting loneliness. A smaller proportion exhibited transient loneliness during the pandemic period. Under the stricter loneliness threshold, the 2021 peak and the predominance of stable over transitional sequences were preserved, although the ranking of specific sequences changed.

**Conclusions:**

Among respondents retained in this five-wave online panel, loneliness prevalence peaked in 2021, declined in 2022, and remained relatively stable from 2022 to 2024. Stable trajectories were more common than transitional trajectories. These findings provide descriptive evidence on long-term loneliness patterns but should not be interpreted as nationally representative prevalence estimates.

## 1. Introduction

Loneliness has received increasing public health attention in recent years [1–4], particularly during the COVID-19 pandemic [5–9]. Social distancing measures, mobility restrictions, and changes in daily routines raised concerns about potential increases in social isolation and subjective loneliness across populations [1,3]. Numerous cross-sectional studies documented elevated levels of loneliness during the early stages of the pandemic [10–12]. However, less is known about how loneliness evolved over multiple years as societies adapted to changing conditions.

Most existing studies rely on cross-sectional data or short-term longitudinal follow-up periods [13–17]. While such studies provide valuable snapshots, they offer limited insight into longer-term stability and change. Understanding whether loneliness levels returned to pre-pandemic patterns, remained elevated, or exhibited heterogeneous trajectories across individuals requires longitudinal data spanning multiple years.

Japan provides a useful setting for examining long-term changes in loneliness because the same nationwide online survey was conducted annually over five consecutive years spanning February 2020 to March 2024. This repeated measurement makes it possible to examine not only annual prevalence but also individual-level stability and transition patterns across the different phases of the COVID-19 period.

The present study uses data from a nationwide online panel survey conducted annually from 2020 to 2024 to describe changes in loneliness over time. Rather than focusing on causal explanations, we document annual prevalence trends and summarize five-year response sequences to characterize common longitudinal patterns. By providing descriptive evidence on both stability and transition in loneliness, this study contributes to ongoing public health monitoring efforts and offers a longitudinal perspective on experiences among respondents retained in the longitudinal panel during and after the COVID-19 period.

## 2. Data and methodology

### 2.1. Data

This study used data from five consecutive waves of the Household Behavioral and Financial Survey conducted by Hiroshima University in 2020, 2021, 2022, 2023, and 2024. The surveys were administered online by Nikkei Research using its own and allied registered monitor panels. Participants were adults aged 20 years or older residing throughout Japan. The survey protocol was reviewed internally by Nikkei Research in accordance with its ethical and survey administration procedures. All participants provided informed consent before completing the questionnaire. The baseline survey was conducted from February 20–25, 2020. The authors requested that the survey company collect approximately 17,000 completed responses. Nikkei Research distributed email invitations to registered panel members until the target sample size was reached, yielding 17,463 effective responses. Because the number of invitations required to reach the target sample was not available to the authors, the response rate for the baseline survey could not be calculated.

Follow-up surveys were conducted from February 19–26, 2021; February 18–28, 2022; February 22 to March 6, 2023; and February 27 to March 11, 2024. The numbers of distributed invitations and effective completed responses were 16,949 and 6,103 in 2021, 6,103 and 4,281 in 2022, 4,221 and 3,410 in 2023, and 3,486 and 2,929 in 2024, respectively. At later waves, some respondents from the preceding wave could not be contacted because they had withdrawn from the survey company’s monitor panel, while some invited panelists did not complete the follow-up survey. The 2020 survey was conducted shortly before the World Health Organization (WHO) declared COVID-19 a pandemic on March 11, 2020 [18]. The 2021–2023 waves were conducted during the pandemic period. The 2024 wave was conducted after the WHO determined on May 5, 2023 that COVID-19 no longer constituted a Public Health Emergency of International Concern [19].

After respondents were matched across all five waves, 2,817 individuals remained in the merged longitudinal dataset. We subsequently excluded observations with missing information on variables required for the analysis, including employment status, financial literacy, household income, and household assets. The final analytic sample consisted of 1,673 respondents. The reduction from the matched five-wave sample to the final analytic sample resulted from complete-case exclusion. Gender, rural residence, university education, and the presence of children were obtained from the 2020 baseline wave. Age and current socioeconomic and psychological characteristics were measured using the 2024 wave. Variables indicating changes in living arrangements, employment, financial conditions, health, future anxiety, financial satisfaction, and depression were constructed using information from 2020 and 2024.

To assess potential selection associated with attrition and complete-case restriction, we compared baseline characteristics between respondents included and not included in the final analytic sample (S1 Appendix). The groups differed in several observed characteristics, including sex, age, university education, financial literacy, and household assets. These differences are considered in the interpretation of the results and discussed as a limitation.

### 2.2. Variables

Loneliness was measured using the three-item UCLA Loneliness Scale developed by Hughes et al. [20]. Participants were asked how often they felt that they lacked companionship, felt left out, and felt isolated from others. Response options were “hardly ever or never,” “some of the time,” and “often.” Participants were classified as lonely if they answered “some of the time” or “often” to at least one of the three items; those who answered “hardly ever or never” to all three items were classified as not lonely. This coding has been used in previous large-scale analyses of Japanese online panel data employing the UCLA-3 items [1,21].

This is an inclusive threshold that captures any reported experience of loneliness at a moderate or higher frequency. It therefore produces higher prevalence estimates than a stricter definition based only on “often” responses. We consequently conducted a sensitivity analysis using the stricter definition (S2 Appendix). Cronbach’s alpha coefficients for the three items ranged from 0.8444 to 0.8637 across the five waves, indicating good internal consistency (S3 Appendix).

Our study incorporated a range of participants’ demographic, socioeconomic, and psychological characteristics to analyze loneliness patterns in greater depth. These variables align with those used in previous research [1,8,21–23].

Gender, rural residence, university education, and the presence of children were measured at baseline in 2020. Age and current socioeconomic and psychological characteristics were measured in 2024. Variables indicating changes in living arrangements, employment, financial conditions, health, future anxiety, financial satisfaction, and depression were constructed using information from 2020 and 2024. The detailed definitions of the variables are provided in Table 1.

**Table 1:** Definitions of variables.

Variables	Definition
Loneliness 2020 Loneliness 2021 Loneliness 2022 Loneliness 2023 Loneliness 2024	Binary variable: 1 = answered “some of the time” or “often” to at least one of the three UCLA-3 items (“How often do you feel you lack companionship?”, “How often do you feel left out?”, and “How often do you feel isolated from others?”); 0 = answered “hardly ever or never” to all three items.
**Demographic and socioeconomic variables**	
Male	Binary variable: 1 = male, 0 = female
Age	Continuous variable: respondents’ age in 2024
Living in rural areas	Binary variable: 1 = rural, 0 = urban
University degree	Binary variable: 1 = education years ≥16, 0 = <16 years of education
Children	Binary variable: 1 = with child, 0 = without child
Living alone	Binary variable: 1 = living alone in 2024, 0 = not living alone in 2024
Full-time employment	Binary variable: 1 = employed full-time in 2024, 0 = not employed full-time in 2024
Financial literacy	Continuous variable: average scores of answers for the three financial literacy questions
Household income	Continuous variable: annual income earned before taxes and with bonuses of the entire household (in JPY)
Log of household income	Log (household income in 2024)
Household asset	Continuous variable: balance of financial assets (savings, stocks, bonds, insurance, etc.) of the entire household (in JPY)
Log of household asset	Log (household asset in 2024)
Financial satisfaction	Ordinal variable for the statement “I am happy with my financial status”; responses: 1 = completely disagree, 2 = disagree, 3 = neutral, 4 = agree, and 5 = completely agree
Subjective health status	Ordinal variable for the statement “I am now healthy and was generally healthy in the last one year”; responses: 1 = not true at all, 2 = not so true, 3 = neutral, 4 = somewhat true, and 5 = true
Future anxiety	Ordinal variable for the statement: “I have anxieties about my life after I am 65 years old” (For those who are already aged 65 or above, “life in future”);responses: 1 = not true at all, 2 = not so true, 3 = neutral, 4 = somewhat true, and 5 =true
Depression	Ordinal variable for the statement “I often feel depressed or have felt depressed in the last 1 year”; responses: 1 = not true at all, 2 = not so true, 3 = neutral, 4 = somewhat true, and 5 = true
No longer had a spouse	Binary variable: 1 = had a spouse in 2020 but did not have a spouse in 2024, 0 = otherwise
Started living alone	Binary variable: 1 = did not live alone in 2020 but lived alone in 2024, 0 = otherwise
Left full-time employment	Binary variable: 1 = employed full-time in 2020 but not employed full-time in 2024, 0 = otherwise
Change in log household income	Log ratio of household income in 2024 to household income in 2020: log(household income in 2024 / household income in 2020)
Change in log household assets	Log ratio of household assets in 2024 to household assets in 2020: log(household assets in 2024 / household assets in 2020)
Change in health status	Binary variable: 1 = subjective health status was lower in 2024 than in 2020, 0 = otherwise
Change in future anxiety	Binary variable: 1 = future anxiety was higher in 2024 than in 2020, 0 = otherwise
Change in financial satisfaction	Binary variable: 1 = financial satisfaction was lower in 2024 than in 2020, 0 = otherwise
Change in depression	Binary variable: 1 = depression was higher in 2024 than in 2020, 0 = otherwise

### 2.3. Methods

We used univariate and bivariate descriptive statistics to examine annual loneliness prevalence and five-year transition patterns. Each respondent was classified as lonely or not lonely in each wave, producing 32 possible five-year response sequences. We report the ten most frequent sequences and summarize broader patterns, including loneliness in all five waves, no loneliness in any wave, onset, and recovery.

To examine associations between the five most frequent loneliness sequences and categorical sociodemographic or psychological characteristics, we created a binary indicator for membership in each sequence and conducted Pearson’s chi-square tests comparing respondents belonging to the sequence with all other respondents.

All descriptive analyses were unweighted. No multivariable regression analysis was performed, and the findings are interpreted descriptively rather than causally.

As a sensitivity analysis, we applied a stricter loneliness threshold, classifying respondents as lonely only if they reported feeling lonely “often” on at least one of the three UCLA items. We then recalculated annual prevalence rates and five-year sequence distributions to assess the sensitivity of the descriptive findings to the operational definition of loneliness.

## 3. Results

### 3.1. Loneliness transitions over the years

Table 2 presents annual loneliness prevalence among the 1,673 respondents included in the final analytic sample. As shown in Table 2, loneliness prevalence peaked in 2021 and was lower and relatively stable from 2022 to 2024.

**Table 2:** Annual prevalence of loneliness from 2020 to 2024.

Variables	n	% (N = 1,673)
Loneliness 2020	1,146	68.50
Loneliness 2021	1,208	72.21
Loneliness 2022	1,076	64.32
Loneliness 2023	1,092	65.27
Loneliness 2024	1,092	65.27

After examining annual loneliness prevalence, we tracked respondents’ loneliness status across the five survey waves. Because loneliness was coded as a binary variable in each wave, 32 possible five-year response sequences were observed. We report the ten most frequent sequences to summarize the predominant patterns in the analytic sample. Table 3 presents the distribution of loneliness, revealing the number and percentage of participants belonging to the most prevalent patterns.

**Table 3:** Top ten most common patterns of loneliness (2020–2024).

No.	Trajectory	n	%
1	Lonely in all five waves	754	45.1%
2	Not lonely in any wave	235	14.0%
3	Lonely in 2020, 2021, 2023, and 2024	57	3.4%
4	Not lonely in 2020; lonely from 2021 to 2024	54	3.2%
5	Lonely in 2020–2022 and 2024	51	3.0%
6	Lonely in 2020–2023; not lonely in 2024	47	2.8%
7	Lonely only in 2021	44	2.6%
8	Lonely only in 2020	39	2.3%
9	Lonely only in 2020 and 2021	33	2.0%
10	Lonely in 2020, 2021, and 2023	29	1.7%

The ten most frequent sequences accounted for 80.3% of the analytic sample. The most common sequence was persistent loneliness across all five waves (11111; 45.1%), followed by no loneliness in any wave (00000; 14.0%). Each of the remaining sequences accounted for 3.4% or less of the sample. Overall, stable sequences were more common than transitional sequences, although several less frequent patterns indicated onset or recovery at different points during the study period.

### 3.2. Descriptive statistics of sociodemographic variables

Table 4 summarizes the characteristics of the final analytic sample. Of the 1,673 respondents, 71.1% were male, and the median age in 2024 was 57 years (IQR 49–66). Approximately 54.9% lived in rural areas, 64.7% had a university degree, 60.1% had children, and 21.1% lived alone. The male-skewed composition of the sample and its implications for generalizability are addressed in the Discussion.

**Table 4:** Characteristics of the final analytic sample (N = 1,673).

Variables	Descriptive statistics	
**Sociodemographic variables**		
Gender	Female	484 (28.93)
	Male	1,189 (71.07)
Rural	Living in rural areas	919 (54.93)
	Otherwise	754 (45.07)
University degree	Having at least a university degree	1,083 (64.73)
	Otherwise	590 (35.27)
Children	Having children	1,005 (60.07)
	Otherwise	668 (39.93)
Living alone	Living alone	353 (21.10)
	Otherwise	1,320 (78.90)
Full-time employment	Having full-time employment	1,019 (60.91)
	Otherwise	654 (39.09)
Financial satisfaction	Completely disagree	259 (15.48)
	Disagree	366 (21.88)
	Neutral	538 (32.16)
	Agree	449 (26.84)
	Completely agree	61 (3.65)
Subjective health status (healthy)	Not true at all	145 (8.67)
	Not so true	308 (18.41)
	Neutral	624 (37.30)
	Somewhat true	421 (25.16)
	True	175 (10.46)
Future anxiety	Not true at all	67 (4.00)
	Not so true	197 (11.78)
	Neutral	334 (19.96)
	Somewhat true	500 (29.89)
	True	575 (34.37)
Depression	Not true at all	268 (16.02)
	Not so true	454 (27.14)
	Neutral	481 (28.75)
	Somewhat true	261 (15.60)
	True	209 (12.49)
Age		57 [49, 66]
Financial literacy		0.73 ± 0.32
Household income (JPY)		5,000,000 [3,000,000; 9,000,000]
Log of household income		15.45 ± 0.80
Household asset (JPY)		12,500,000 [3,750,000; 40,000,000]
Log of household asset		16.22 ± 1.45
**Changes in sociodemographic variables**		
No longer had a spouse	Yes	36 (2.15)
	No	1,637 (97.85)
Started living alone	Yes	54 (3.23)
	No	1,619 (96.77)
Left full-time employment	Yes	104 (6.22)
	No	1,569 (93.78)
Change in health status	Yes	516 (30.84)
	No	1,157 (69.16)
Change in future anxiety	Yes	482 (28.81)
	No	1,191 (71.19)
Change in financial satisfaction	Yes	388 (23.19)
	No	1,285 (76.81)
Change in depression	Yes	457 (27.32)
	No	1,216 (72.68)
Change in log household income		−0.02 ± 0.5
Change in log household assets		0.16 ± 0.75
Observations		1,673

**Note:** We report n (%) for categorical variables, median [IQR] for age, household income, and household assets, and mean ± SD for financial literacy, log of household income, log of household asset, Change in log household income, and Change in log household assets.

### 3.3. Chi-square test results

Table 5 presents the distribution of the top five loneliness patterns by various sociodemographic and psychological variables.

**Table 5:** Chi-square analysis of sociodemographic and psychological characteristics across the top five loneliness patterns.

Variables		Top patterns														
		11111 (n=754)			00000 (n=235)			11011 (n=57)			01111 (n=54)			11101 (n=51)		
		No	Yes	χ²	No	Yes	χ²	No	Yes	χ²	No	Yes	χ²	No	Yes	χ²
Male	No	257	227	0.92	425	59	1.94	472	12	1.78	463	21	2.69	470	14	0.06
	Yes	662	527		1,013	176		1,144	45		1,156	33		1,152	37	
Old age	No	583	602	53.93**	1,063	122	47.35**	1,144	41	0.03	1,143	42	1.3	1,143	42	3.38
	Yes	336	152		375	113		472	16		476	12		479	9	
Living in rural areas	No	427	327	1.6	642	112	0.74	732	22	1	725	29	1.68	730	24	0.08
	Yes	492	427		796	123		884	35		894	25		892	27	
Children	No	307	361	36.17**	611	57	28.00**	640	28	2.08	648	20	0.19	648	20	0.01
	Yes	612	393		827	178		976	29		971	34		974	31	
University degree	No	304	286	4.27*	525	65	6.93**	567	23	0.67	572	18	0.09	575	15	0.79
	Yes	615	468		913	170		1,049	34		1,047	36		1,047	36	
No longer had a spouse	No	900	737	0.07	1,406	231	0.26	1,581	56	0.04	1,585	52	0.64	1,587	50	0.01
	Yes	19	17		32	4		35	1		34	2		35	1	
Started living alone	No	893	726	1.04	1,389	230	1.06	1,564	55	0.01	1,568	51	0.97	1,570	49	0.08
	Yes	26	28		49	5		52	2		51	3		52	2	
Left full-time employment	No	867	702	1.09	1,351	218	0.49	1,514	55	0.74	1,518	51	0.04	1,519	50	1.63
	Yes	52	52		87	17		102	2		101	3		103	1	
Change in health status	No	639	518	0.13	990	167	0.47	1,114	43	1.09	1,123	34	1	1,124	33	0.49
	Yes	280	236		448	68		502	14		496	20		498	18	
Change in future anxiety	No	653	538	0.02	1,021	170	0.18	1,150	41	0.02	1,158	33	2.76	1,157	34	0.52
	Yes	266	216		417	65		466	16		461	21		465	17	
Change in financial satisfaction	No	718	567	2	1,103	182	0.06	1,242	43	0.06	1,246	39	0.66	1,245	40	0.08
	Yes	201	187		335	53		374	14		373	15		377	11	
Change in depression	No	684	532	3.13	1,041	175	0.44	1,173	43	0.23	1,185	31	6.56*	1,181	35	0.44
	Yes	235	222		397	60		443	14		434	23		441	16	

**Note:** In each five-digit code, 1 indicates loneliness and 0 indicates no loneliness, in chronological order from 2020 to 2024. Old age indicates age ≥65 years. *p < 0.05; ** p < 0.01.

#### 3.3.1. Long-term loneliness.

Membership in the persistent-loneliness sequence (lonely in all five waves) differed significantly by age, having children, and university education. Persistent loneliness was more common among respondents younger than 65 years (50.8%; 602/1,185) than among those aged 65 years or older (31.1%; 152/488; χ² = 53.93, p < 0.01). It was also more common among respondents without children (54.0%; 361/668) than among those with children (39.1%; 393/1,005; χ² = 36.17, p < 0.01). The sequence was slightly more common among respondents without a university degree (48.5%; 286/590) than among those with a university degree (43.2%; 468/1,083; χ² = 4.27, p < 0.05).

#### 3.3.2. No loneliness in any wave.

Membership in the “not lonely in any wave” sequence also differed by age, having children, and university education. The sequence was more common among respondents aged 65 years or older (23.2%; 113/488) than among those younger than 65 years (10.3%; 122/1,185; χ² = 47.35, p < 0.01). It was more common among respondents with children (17.7%; 178/1,005) than among those without children (8.5%; 57/668; χ² = 28.00, p < 0.01), and among respondents with a university degree (15.7%; 170/1,083) than among those without a university degree (11.0%; 65/590; χ² = 6.93, p < 0.01).

#### 3.3.3. Fluctuating but long-term loneliness.

Associations were less consistent for the smaller trajectory groups. Some descriptive differences were observed, including a relatively high proportion of worsening depression in the “not lonely in 2020; lonely from 2021 to 2024” group, but these findings should be interpreted cautiously because of the small number of respondents in each sequence.

### 3.4. Sensitivity analysis

Under the stricter definition, in which respondents were classified as lonely only if they answered “often” to at least one of the three UCLA items, annual loneliness prevalence was 28.2% in 2020, 32.0% in 2021, 28.1% in 2022, 25.5% in 2023, and 26.7% in 2024. Thus, the stricter threshold substantially reduced the absolute prevalence estimates, although prevalence still peaked in 2021 and was lower in all subsequent waves.

The distribution of specific five-year sequences also differed from that obtained using the primary definition. Under the stricter definition, the most common sequence was no loneliness in any wave (Not lonely in any wave; 50.4%), followed by loneliness in all five waves (Lonely in all five waves; 11.3%). Nevertheless, under both definitions, stable sequences—either loneliness in all five waves or no loneliness in any wave—were more common in combination than the remaining transitional sequences. Full sensitivity-analysis results are presented in S2 Appendix.

## 4. Discussion

This study provides descriptive evidence on five-year loneliness trajectories in Japan from 2020 to 2024 using a nationwide online panel. Rather than focusing on causal explanations, we documented annual prevalence trends and longitudinal response patterns to better understand how loneliness evolved over the course of the COVID-19 period and its aftermath.

The annual prevalence of loneliness peaked in 2021, declined in 2022, and remained relatively stable from 2022 to 2024. While temporary fluctuations were observed during the early pandemic period, the overall pattern did not suggest a sustained escalation over the five-year span. These descriptive patterns are similar to reports from other settings indicating short-term disruptions followed by partial stabilization [8,17,24].

The predominance of stable sequences indicates that loneliness patterns did not change uniformly across respondents. The two stable sequences—loneliness in all five waves and no loneliness in any wave—together accounted for most respondents, whereas onset and recovery sequences were individually much less common. This heterogeneity cautions against interpreting changes in annual prevalence as evidence that the same individuals uniformly became more or less lonely.

The annual pattern observed in this study is broadly consistent with earlier evidence showing that loneliness worsened during the early phase of the pandemic, although later trajectories varied across studies and populations. In Japan, the JACSIS 2020–2021 study reported an increase in loneliness between the first and second pandemic years [12], while a separate two-year longitudinal study found that loneliness and social isolation remained unresolved across prolonged pandemic phases [17]. In the Swiss Household Panel, increases in loneliness were disproportionately concentrated among younger respondents [16]. Our findings extend this evidence by covering five annual waves and suggest that, among respondents retained in the present panel, the increase observed in 2021 was followed by lower and relatively stable prevalence from 2022 to 2024.

Longitudinal trajectory analysis indicated that most respondents demonstrated stable patterns across the five waves, either consistently reporting loneliness or consistently not reporting loneliness. A smaller proportion exhibited transitional patterns, including temporary increases or decreases during the study period. These descriptive patterns highlight heterogeneity in lived experiences during and after the pandemic.

Descriptive comparisons across sociodemographic and psychological characteristics did not reveal uniform or consistent distributional differences across trajectory groups. While certain characteristics appeared more common in some trajectory categories, no single factor clearly distinguished all patterns. These observations underscore the complexity of loneliness as a multidimensional and context-dependent experience.

By following the same respondents over five annual waves, this study provides a longer-term perspective than a single cross-sectional snapshot. Monitoring stability and transition patterns over time may be important for identifying groups that experience persistent loneliness, while recognizing that many individuals demonstrate stable trajectories.

Several limitations should be acknowledged. First, loneliness was measured using a brief three-item scale and dichotomized using an inclusive threshold. This operationalization captured respondents who endorsed any item at least “some of the time” and consequently produced relatively high prevalence estimates. The sensitivity analysis using a stricter threshold yielded substantially lower prevalence, although the annual peak in 2021 and the predominance of stable over transitional sequences remained.

Second, attrition across the five survey waves was substantial. The baseline comparison indicated that respondents included in the final analytic sample differed from those not included in several observed characteristics. Included respondents were older, more likely to be male and university educated, and had higher financial literacy and household assets. These differences indicate potential selection bias, and unobserved differences may also remain. The findings should therefore be interpreted as descriptive patterns among retained respondents rather than as nationally representative prevalence estimates.

Third, participation required continued access to and engagement with an online survey panel. Older adults who participate repeatedly in online surveys may differ from older adults with limited internet access or lower digital familiarity. They may also differ in health status, socioeconomic resources, and their capacity to participate in repeated surveys. Consequently, the age-related patterns observed in this study may not generalize to the broader older population in Japan.

Fourth, the analytic sample was substantially male-skewed, with men accounting for 71.1% of respondents. Because loneliness patterns may differ by sex, this imbalance may have influenced both prevalence estimates and trajectory distributions.

Fifth, the baseline response rate could not be calculated because the survey company continued recruitment until the target sample size was reached and the total number of invitations was unavailable to the authors. Finally, the analyses were descriptive and did not establish causal relationships between the COVID-19 period, individual characteristics, and loneliness trajectories.

Despite these limitations, the study offers five-year panel evidence on loneliness trajectories in Japan. Future research may build upon these descriptive findings using analytical approaches designed to examine potential mechanisms and causal pathways.

## 5. Conclusions

This study examined annual prevalence and five-year transition patterns of loneliness among respondents retained in a nationwide Japanese online panel from 2020 to 2024. Loneliness prevalence peaked in 2021, declined in 2022, and remained relatively stable from 2022 to 2024.

Loneliness in all five waves and no loneliness in any wave were the two most common sequences, while onset and recovery patterns were less frequent. These findings indicate substantial heterogeneity in individual loneliness experiences across the study period.

Because of attrition, the online survey mode, and the male-skewed analytic sample, the results should not be interpreted as nationally representative prevalence estimates. Nevertheless, the five-wave data provide descriptive evidence on the stability and transition of loneliness over an extended period spanning the COVID-19 emergency and its aftermath.

## Supporting information

S1 Appendix Baseline characteristics of participants included and not included in the final analytic sample. (DOCX)

S2 Appendix Sensitivity analysis. (DOCX)

S3 Appendix Cronbach’s alpha for the UCLA-3 items. (DOCX)

S1 Data Minimal anonymized dataset necessary to replicate the findings reported in this study. (DTA)
