# Supplementary material for: The dynamics of loneliness: A longitudinal exploration of transitions in Japan across the COVID-19 period, 2020–2024
Source: PLoS One. 2026 Sep 11;21(9):e0358269. doi: 10.1371/journal.pone.0358269 (PMC13567814; doi:10.1371/journal.pone.0358269)
Supplement: Supplementary file 1 [file pone.0358269.s001.docx]

**S1 Appendix**. Baseline characteristics of participants included and not included in the final analytic sample.

| **Variables** | | **Excluded from the final analytic sample (2020)** | **Included in the final analytic sample** | **Test statistics** | **SMD*** |
| --- | --- | --- | --- | --- | --- |
| Gender | Female | 7,203 (45.62) | 484 (28.93) | 170.9452*** | -0.35 |
|  | Male | 8,587 (54.38) | 1,189 (71.07) |  |  |
| Rural | Living in rural areas | 9,821 (62.2) | 919 (54.93) | 33.7334*** | 0.15 |
|  | Otherwise | 5,969 (37.8) | 754 (45.07) |  |  |
| University degree | Having at least a university degree | 8,680 (54.97) | 1,083 (64.73) | 54.4852*** | -0.20 |
|  | Otherwise | 7,110 (45.03) | 590 (35.27) |  |  |
| Children | Having children | 8,492 (53.78) | 1,005 (60.07) | 24.1318*** | -0.13 |
|  | Otherwise | 7,298 (46.22) | 668 (39.93) |  |  |
| Living alone | Living alone | 2,635 (16.69) | 324 (19.37) | 7.7124*** | -0.07 |
|  | Otherwise | 13,155 (83.31) | 1,349 (80.63) |  |  |
| Full-time employment | Having full-time employment | 9,726 (61.6) | 1,082 (64.47) | 6.0775** | -0.06 |
|  | Otherwise | 6,064 (38.4) | 591 (35.33) |  |  |
| Financial satisfaction | Completely disagree | 2,653 (16.8) | 227 (13.57) | 22.8318*** | -0.12 |
|  | Disagree | 4,193 (26.55) | 429 (25.64) |  |  |
|  | Neutral | 5,251 (33.26) | 554 (33.11) |  |  |
|  | Agree | 3,165 (20.04) | 389 (23.25) |  |  |
|  | Completely agree | 528 (3.34) | 74 (4.42) |  |  |
| Subjective health status (healthy) | Not true at all | 1,102 (6.98) | 117 (6.99) | 6.7906 | 0.03 |
|  | Not so true | 2,562 (16.23) | 292 (17.45) |  |  |
|  | Neutral | 6,091 (38.58) | 634 (37.90) |  |  |
|  | Somewhat true | 3,854 (24.41) | 431 (25.76) |  |  |
|  | True | 2,181 (13.81) | 199 (11.89) |  |  |
| Future anxiety | Not true at all | 797 (5.05) | 97 (5.80) | 36.838*** | 0.10 |
|  | Not so true | 1,444 (9.15) | 215 (12.85) |  |  |
|  | Neutral | 3,964 (25.10) | 384 (22.95) |  |  |
|  | Somewhat true | 4,363 (27.63) | 495 (29.59) |  |  |
|  | True | 5,222 (33.07) | 482 (28.81) |  |  |
| Depression | Not true at all | 2,018 (12.78) | 238 (14.23) | 39.1229*** | 0.14 |
|  | Not so true | 3,278 (20.76) | 438 (26.18) |  |  |
|  | Neutral | 5,258 (33.30) | 530 (31.68) |  |  |
|  | Somewhat true | 3,093 (19.59) | 292 (17.45) |  |  |
|  | True | 2,143 (13.57) | 175 (10.46) |  |  |
| Age | | 46 [34, 57] | 53 [45, 62] | -18.8142*** | -0.52 |
| Financial literacy | | 0.561 ± 0.386 | 0.73 ± 0.321 | -17.1498*** | -0.47 |
| Household income (Unit: JPY) | | 5,000,000 [3,000,000; 9,000,000] | 5,000,000 [3,000,000; 9,000,000] | -2.4814** | -0.06 |
| Household asset (Unit: JPY) | | 6,250,000 [1,250,000; 17,500,000] | 12,500,000 [3,750,000; 25,000,000] | -11.2080*** | -0.27 |
| Observations | | 15,790 | 1,673 | - | - |
| **Note:** We report n (%) for categorical variables, median [IQR] for age, household income, and household assets, and mean ± SD for the continuous variable of financial literacy. Pearson's chi-square tests were used for categorical variables and independent samples t-tests for continuous variables. SMD: standardized mean differences. ***p-values <0.01, **p-values<0.05 | | | | | |
