# Supplementary material for: The dynamics of loneliness: A longitudinal exploration of transitions in Japan across the COVID-19 period, 2020–2024
Source: PLoS One. 2026 Sep 11;21(9):e0358269. doi: 10.1371/journal.pone.0358269 (PMC13567814; doi:10.1371/journal.pone.0358269)
Supplement: Supplementary file 2 [file pone.0358269.s002.docx]

**S2 Appendix.** Sensitivity analysis.

| **Primary results**  where respondents are classified as lonely if they answered “some of the time” or “often” to at least one UCLA-3 item  Trajectory \| n \| % | | | **Results using an alternative loneliness measure** where respondents are classified as lonely if they answered “often” to at least one UCLA-3 item  Trajectory \| n \| % | | |
| --- | --- | --- | --- | --- | --- |
| **Variables** | **n** | **%**  **(N = 1,673)** | **Variables** | **n** | **%**  **(N= 1,673)** |
| Loneliness 2020 | 1,146 | 68.50 | Loneliness 2020 | 471 | 28.15 |
| Loneliness 2021 | 1,208 | 72.21 | Loneliness 2021 | 536 | 32.04 |
| Loneliness 2022 | 1,076 | 64.32 | Loneliness 2022 | 470 | 28.09 |
| Loneliness 2023 | 1,092 | 65.27 | Loneliness 2023 | 426 | 25.46 |
| Loneliness 2024 | 1,092 | 65.27 | Loneliness 2024 | 447 | 26.72 |
| **Ten most common five-year loneliness trajectories** | | | | | |
| Lonely in all five waves | 754 | 45.07 | Not lonely in any wave | 843 | 50.39 |
| Not lonely in any wave | 235 | 14.05 | Lonely in all five waves | 189 | 11.3 |
| Lonely in 2020, 2021, 2023, and 2024 | 57 | 3.41 | Lonely only in 2021 | 76 | 4.54 |
| Not lonely in 2020; lonely from 2021 to 2024 | 54 | 3.23 | Lonely only in 2020 | 63 | 3.77 |
| Lonely in 2020–2022 and 2024 | 51 | 3.05 | Lonely only in 2024 | 46 | 2.75 |
| Lonely in 2020–2023; not lonely in 2024 | 47 | 2.81 | Lonely only in 2022 | 38 | 2.27 |
| Lonely only in 2021 | 44 | 2.63 | Lonely in 2020–2023; not lonely in 2024 | 35 | 2.09 |
| Lonely only in 2020 | 39 | 2.33 | Lonely only in 2023 | 30 | 1.79 |
| Lonely only in 2020 and 2021 | 33 | 1.97 | Not lonely in 2020; lonely from 2021 to 2024 | 28 | 1.67 |
| Lonely in 2020, 2021, and 2023 | 29 | 1.73 | Lonely only in 2021 and 2022 | 24 | 1.43 |

Note: Trajectories are ranked separately under the primary and alternative loneliness definitions; patterns shown on the same row are not directly matched.
