# Supplementary material for: The dynamics of loneliness: A longitudinal exploration of transitions in Japan across the COVID-19 period, 2020–2024
Source: PLoS One. 2026 Sep 11;21(9):e0358269. doi: 10.1371/journal.pone.0358269 (PMC13567814; doi:10.1371/journal.pone.0358269)
Supplement: Supplementary file 3 [file pone.0358269.s003.docx]

**S3 Appendix.** Cronbach’s alpha for the UCLA-3 items.

|  | **Internal consistency score** |
| --- | --- |
| Loneliness in 2020 | 0.8499 |
| Loneliness in 2021 | 0.8444 |
| Loneliness in 2022 | 0.8568 |
| Loneliness in 2023 | 0.8614 |
| Loneliness in 2024 | 0.8637 |
