# Supplementary material for: The dynamics of loneliness: A longitudinal exploration of transitions in Japan across the COVID-19 period, 2020–2024
Source: PLoS One. 2026 Sep 11;21(9):e0358269. doi: 10.1371/journal.pone.0358269 (PMC13567814; doi:10.1371/journal.pone.0358269)
Supplement: Supplementary file 4 [file pone.0358269.s006.docx]

**Response to Reviewers**

**Manuscript ID:** PONE-D-26-09247
**Title:** *The Dynamics of Loneliness: A Longitudinal Exploration of Transitions in Japan Across the COVID-19 Period, 2020–2024*

Dear Academic Editor and Reviewer 1,

Thank you very much for your careful and constructive evaluation of our manuscript. We have revised the manuscript substantially in response to the comments.

The principal revisions include a more detailed description of the survey administration and participant flow; clarification of the baseline recruitment procedure and follow-up response counts; a new comparison between respondents included and not included in the final analytic sample; expanded discussion of attrition, selection bias, online-panel participation, age-related digital access, and the male-skewed sample; clarification of the UCLA-3 threshold; a sensitivity analysis using a stricter definition of loneliness; expanded comparison with other longitudinal studies; and revised presentation of the descriptive tables.

We have also removed the previous claims concerning representativeness and now interpret the results as descriptive findings among respondents retained in the five-wave online panel.

Our point-by-point responses are provided below.

**Responses to the Academic Editor**

**Comment 1**

Page 16: “loneliness continue to represent an important public health issue,” should read: “loneliness continues to represent an important public health issue.”

**Response:**

Thank you. During revision, we condensed this part of the Results section and removed the sentence containing the grammatical error. The Results now provide a concise description of the annual pattern.

**Comment 2**

Lines 124–125: “This approach is consistent with previous large-scale survey studies using the UCLA-3 scale.” Please cite the studies supporting this statement. This is particularly important because the threshold adopted produced loneliness prevalences ranging from 64% to 72%, which are considerably higher than estimates reported in most international studies.

**Response:**

We agree that the operational definition required clearer explanation and support. We have added citations to relevant previous large-scale Japanese online-panel studies using the UCLA-3 items and the same inclusive coding approach.

We now explain explicitly that the primary definition classifies respondents as lonely when they answer “some of the time” or “often” to at least one of the three UCLA items. We acknowledge that this inclusive threshold produces relatively high prevalence estimates.

We also added a sensitivity analysis using a stricter definition, under which respondents were classified as lonely only if they answered “often” to at least one item. The stricter definition substantially reduced annual prevalence estimates. However, prevalence continued to peak in 2021, and stable trajectories remained more common than transitional trajectories. We also clarify that the distribution and ranking of specific five-year sequences changed under the stricter definition.

These revisions appear in Sections 2.2, 2.3, and 3.4, the Discussion, and Appendix B.

**Comment 3**

Please expand the discussion section. It would benefit from comparisons with findings from other longitudinal cohorts investigating loneliness trajectories during and after the COVID-19 pandemic.

**Response:**

We have expanded the Discussion to compare our findings with repeated nationwide survey evidence from the JACSIS 2020–2021 study, a two-year longitudinal study conducted in Japan, and longitudinal evidence from the Swiss Household Panel.

The revised Discussion explains that previous studies generally identified worsening loneliness during the earlier phase of the pandemic, while subsequent patterns varied across populations. We also clarify that the present study extends this literature by examining five annual waves from 2020 to 2024.

**Comment 4**

The manuscript alternates between “COVID-19 timeline,” “pandemic era,” and “post-pandemic era.” Consider adopting consistent terminology throughout.

**Response:**

We agree. We revised the terminology throughout the body of the manuscript to use more precise descriptions of the study periods. These now include the February 2020 baseline, the pandemic-period waves, and the 2024 follow-up conducted after the WHO determined that COVID-19 no longer constituted a Public Health Emergency of International Concern.

We also removed the previous wording suggesting that the pandemic itself “ended” in 2023.

**Comment 5**

Although the authors acknowledge attrition as a limitation and provide descriptive comparisons before and after exclusion of missing data, additional discussion regarding potential selection bias is important. Individuals who remain in long-term panels may differ systematically from those lost to follow-up. They are often healthier, wealthier, and socially more engaged than those lost to follow-up, potentially influencing estimates of loneliness trajectories. The authors should discuss this issue in greater detail and, if possible, provide comparisons between retained and non-retained participants.

**Response:**

We agree and have made several substantial revisions.

First, we added a comparison of baseline characteristics between respondents included and not included in the final analytic sample. The results are presented in Appendix A and include standardized mean differences.

The comparison showed differences in several observed characteristics. Respondents included in the final analytic sample were older, more likely to be male and university educated, and had higher financial literacy and household assets.

We therefore expanded the Discussion to acknowledge potential selection bias and the possibility of additional unobserved differences. The revised manuscript explicitly states that the findings should be interpreted as descriptive patterns among retained online-panel respondents rather than as nationally representative prevalence estimates.

**Responses to Reviewer 1**

We thank Reviewer 1 for the careful review and constructive comments.

**General comment on the Abstract**

Abstract – I have no comments. The authors are sufficiently cautious, and the overall abstract is consistent with the main paper.

**Response:**

Thank you for this positive assessment. Although no specific revision was required in response to this comment, we updated the Abstract to ensure consistency with the revised Methods, sensitivity analysis, and more cautious interpretation of generalizability.

**Major comments**

**Comment 1**

Description of context and how the survey was conducted is limited.

**Response:**

We agree. Section 2.1 has been substantially expanded.

The revised manuscript now identifies Nikkei Research as the survey administrator; describes the use of its own and allied registered online monitor panels; provides the survey dates for all five waves; explains the baseline target-sample recruitment procedure; and reports the numbers of distributed invitations and effective completed responses for the follow-up waves.

We also clarify that the participants were adults aged 20 years or older residing throughout Japan.

**Comment 2**

The authors do not report how many possible responders were contacted and thus do not report the response rate. This should be done or explain why it is not necessary. The argument that the quality and representativeness of each wave were preserved is insufficient.

**Response:**

Thank you. We have removed the previous statement that the quality and representativeness of each wave were preserved.

For the 2020 baseline survey, the authors requested approximately 17,000 completed responses, and the survey company distributed invitations until the target sample size was reached. The total number of invitations required to reach the target was not available to the authors. Consequently, the baseline response rate could not be calculated. This is now stated explicitly in both the Data section and the limitations.

For the follow-up waves, we now report the numbers of distributed invitations and effective completed responses:

- 2021: 16,949 invitations and 6,103 effective responses
- 2022: 6,103 invitations and 4,281 effective responses
- 2023: 4,221 invitations and 3,410 effective responses
- 2024: 3,486 invitations and 2,929 effective responses

We no longer interpret the analytic sample as nationally representative.

**Comment 3**

The authors do not discuss the reasons for the substantial decline of responders from the first to the second-fifth wave. If I understand correctly, only 1,673 responders participated in all waves and were included in the analysis, while one would expect approximately 6,800 respondents.

**Response:**

We agree that the participant flow required clearer explanation.

The revised Data section now distinguishes among the effective responses collected at each wave, the matched five-wave sample, and the final complete-case analytic sample. The 2024 survey yielded 2,929 effective completed responses. After matching respondents across all five waves, 2,817 individuals remained. After excluding observations with missing information on variables required for the analysis, the final analytic sample consisted of 1,673 respondents.

We also explain that some respondents could not be invited to later waves because they had withdrawn from the survey company’s monitor panel. Noncompletion among those invited and complete-case exclusion also contributed to the reduction in sample size.

The sum of the wave-specific sample counts does not represent the number of distinct individuals, because respondents could participate in multiple waves. The final analysis required the same individual to be matched across all five waves and to have complete information on the variables used.

The implications of the resulting attrition and complete-case restriction are now discussed explicitly, and Appendix A compares respondents included and not included in the final analytic sample.

**Comment 4**

The authors do not discuss age as a limitation for participating in an online survey and how age could be a source of bias. What characterizes the older participants in the dataset? Do they differ from typical Japanese older adults, given that they are online users?

**Response:**

We agree that continued participation in an online panel may select a particular group of older adults.

We added a specific limitation explaining that older adults who repeatedly participate in online surveys may differ from older adults with limited internet access or lower digital familiarity. They may also differ in their health, socioeconomic resources, and capacity to participate in repeated surveys.

The present dataset does not permit a direct comparison between the older participants and the entire older population of Japan. We therefore do not claim that the older respondents are representative of all older Japanese adults. Instead, the revised manuscript states that the age-related patterns may not generalize to the broader older population.

**Comment 5**

The authors do not discuss the skewed sex distribution. Approximately 71% of the respondents were male, which threatens the representativeness argument. This should be discussed in the Discussion section.

**Response:**

We agree. We removed the previous claims concerning representativeness and added a separate limitation addressing the male-skewed analytic sample.

The revised manuscript states that men accounted for 71.1% of the final analytic sample and acknowledges that this imbalance may have influenced both the prevalence estimates and trajectory distributions. We therefore interpret the results as descriptive findings among retained panel respondents rather than as nationally representative estimates.

**Minor comments**

**Comment 6**

Lines 61–63: The particular relevance of examining loneliness in Japan should not be based on social isolation, demographic aging, and changing household structures, because these apply to many countries. Annual mapping may be a more relevant argument.

**Response:**

We agree. We replaced the previous justification with a more specific rationale.

The revised Introduction now explains that Japan provides a useful setting because the same nationwide online survey was conducted annually over five consecutive years, enabling assessment of both annual prevalence and individual-level stability and transition patterns.

**Comment 7**

Line 127: This information and reference are already presented and could be omitted.

**Response:**

We removed the redundant description and consolidated the explanation of the UCLA-3 measure, its coding, and its internal consistency in Section 2.2.

**Comment 8**

Lines 188–190: The word “slightly” may be misleading when describing the decline from 72.2% to 64.3%.

**Response:**

We agree. The word “slightly” and the original sentence were removed. The Results now state concisely that loneliness prevalence peaked in 2021 and was lower and relatively stable from 2022 to 2024.

**Comment 9**

Table 2: Consider presenting mean values as percentages, and results should not be repeated in both the table and the text.

**Response:**

Table 2 has been reformatted to present the number and percentage of respondents classified as lonely in each year.

We also shortened the accompanying text so that it summarizes only the principal pattern instead of repeating every value reported in the table.

**Comment 10**

The comparison between 3.2% who remained lonely and 2.6% who recovered should be supported by a significance test; otherwise, it should not be reported.

**Response:**

We agree. The comparison and the word “Interestingly” were removed. We now describe the less frequent trajectories without claiming that the difference between 3.2% and 2.6% is statistically meaningful.

**Comment 11**

As each variable is dichotomized, is min-max in Table 2 necessary? This also applies to Table 4.

**Response:**

We agree. Minimum and maximum values have been removed for dichotomous variables.

Table 2 now reports counts and percentages. Table 4 reports categorical variables as n (%) and continuous variables using appropriate descriptive statistics.

**Comment 12**

Table 4: Consider presenting means as percentages. Age does not need three decimal places.

**Response:**

Table 4 has been revised accordingly. Categorical variables are now presented as n (%), and age is no longer reported to three decimal places.

**Comment 13**

Table 4: Consider presenting median age in case the age distribution is skewed.

**Response:**

We agree. Table 4 now reports the median age and interquartile range. The median age in 2024 was 57 years, with an interquartile range of 49–66 years.

We again thank the Academic Editor and Reviewer 1 for their thoughtful comments. We believe that the revisions have improved the transparency, precision, and interpretation of the manuscript.

Sincerely,

**Yoshihiko Kadoya**
Corresponding Author
On behalf of all authors
