# Supplementary material for: The dynamics of loneliness: A longitudinal exploration of transitions in Japan across the COVID-19 period, 2020–2024
Source: PLoS One. 2026 Sep 11;21(9):e0358269. doi: 10.1371/journal.pone.0358269 (PMC13567814; doi:10.1371/journal.pone.0358269)
Supplement: Peer review history

# Peer review: Decision Letter 0

**DOI:** 10.1371/journal.pone.0358269.r001

**Authors:** Bruna Luchesi

1 Jul 2026

Dear Dr. Khan,

Thank you for submitting your manuscript to PLOS ONE. After careful consideration, we feel that it has merit but does not fully meet PLOS ONE’s publication criteria as it currently stands. Therefore, we invite you to submit a revised version of the manuscript that addresses the points raised during the review process.

==============================

This manuscript addresses an important public health issue using a valuable five-wave longitudinal dataset from Japan. The study is methodologically transparent and well aligned with the aims and scope of PLOS ONE. However, clarification and further discussion are necessary in some parts of the manuscript. Therefore, I recommend major revision.

==============================

Please submit your revised manuscript by Aug 15 2026 11:59PM. If you will need more time than this to complete your revisions, please reply to this message or contact the journal office at plosone@plos.org. When you're ready to submit your revision, log on to https://www.editorialmanager.com/pone/ and select the 'Submissions Needing Revision' folder to locate your manuscript file.

- A letter that responds to each point raised by the academic editor and reviewer(s). You should upload this letter as a separate file labeled 'Response to Reviewers'.
- A marked-up copy of your manuscript that highlights changes made to the original version. You should upload this as a separate file labeled 'Revised Manuscript with Track Changes'.
- An unmarked version of your revised paper without tracked changes. You should upload this as a separate file labeled 'Manuscript'.

If you would like to make changes to your financial disclosure, please include your updated statement in your cover letter. Guidelines for resubmitting your figure files are available below the reviewer comments at the end of this letter.

If applicable, we recommend that you deposit your laboratory protocols in protocols.io to enhance the reproducibility of your results. Protocols.io assigns your protocol its own identifier (DOI) so that it can be cited independently in the future. For instructions see: https://journals.plos.org/plosone/s/submission-guidelines#loc-laboratory-protocols. Additionally, PLOS ONE offers an option for publishing peer-reviewed Lab Protocol articles, which describe protocols hosted on protocols.io. Read more information on sharing protocols at https://plos.org/protocols?utm_medium=editorial-email&utm_source=authorletters&utm_campaign=protocols.

As the corresponding author, your ORCID iD is verified in the submission system and will appear in the published article. PLOS supports the use of ORCID, and we encourage all coauthors to register for an ORCID iD and use it as well. Please encourage your coauthors to verify their ORCID iD within the submission system before final acceptance, as unverified ORCID iDs will not appear in the published article. *Only*  the individual author can complete the verification step; PLOS staff *cannot*  verify ORCID iDs on behalf of authors.

We look forward to receiving your revised manuscript.

Kind regards,

Bruna Moretti Luchesi, Ph.D.

Academic Editor

PLOS One

**Journal Requirements:**

1. When submitting your revision, we need you to address these additional requirements.

Please ensure that your manuscript meets PLOS ONE's style requirements, including those for file naming. The PLOS ONE style templates can be found at

https://journals.plos.org/plosone/s/file?id=wjVg/PLOSOne_formatting_sample_main_body.pdf and

https://journals.plos.org/plosone/s/file?id=ba62/PLOSOne_formatting_sample_title_authors_affiliations.pdf

2. We note that the grant information you provided in the ‘Funding Information’ and ‘Financial Disclosure’ sections do not match.

When you resubmit, please ensure that you provide the correct grant numbers for the awards you received for your study in the ‘Funding Information’ section.

3. Thank you for stating in your Funding Statement:

This work was supported by Rakuten Securities (awarded to Y.K.), the Ministry of Health, Labour and Welfare of Japan under the Health and Labour Sciences Research Grant (Grant Number: 25GB1002) (awarded to Yoshihiko Kadoya), and JSPS KAKENHI through grant numbers JP23K25534, 24K21417 (awarded to Y.K.), and JP23K12503 (awarded to M.S.R.K.). Rakuten Securities (https://www.rakuten-sec.co.jp) (accessed on 28 November 2024), the Ministry of Health, Labour and Welfare of Japan (https://www.mhlw.go.jp/english/) (Accessed on 24 February 2026), and JSPS KAKENHI (https://www.jsps.go.jp/english/e-grants/) (accessed on 28 November 2024) played no role in the study design, analysis, manuscript preparation, or publishing decision.

Please provide an amended statement that declares *all* the funding or sources of support (whether external or internal to your organization) received during this study, as detailed online in our guide for authors at http://journals.plos.org/plosone/s/submit-now.  Please also include the statement “There was no additional external funding received for this study.” in your updated Funding Statement.

Please include your amended Funding Statement within your cover letter. We will change the online submission form on your behalf.

4. We note that you have indicated that there are restrictions to data sharing for this study. For studies involving human research participant data or other sensitive data, we encourage authors to share de-identified or anonymized data. However, when data cannot be publicly shared for ethical reasons, we allow authors to make their data sets available upon request. For information on unacceptable data access restrictions, please see http://journals.plos.org/plosone/s/data-availability#loc-unacceptable-data-access-restrictions.

Before we proceed with your manuscript, please address the following prompts:

a) If there are ethical or legal restrictions on sharing a de-identified data set, please explain them in detail (e.g., data contain potentially identifying or sensitive patient information, data are owned by a third-party organization, etc.) and who has imposed them (e.g., a Research Ethics Committee or Institutional Review Board, etc.). Please also provide contact information for a data access committee, ethics committee, or other institutional body to which data requests may be sent.

b) If there are no restrictions, please upload the minimal anonymized data set necessary to replicate your study findings to a stable, public repository and provide us with the relevant URLs, DOIs, or accession numbers. Please see http://www.bmj.com/content/340/bmj.c181.long for guidelines on how to de-identify and prepare clinical data for publication. For a list of recommended repositories, please see https://journals.plos.org/plosone/s/recommended-repositories. You also have the option of uploading the data as Supporting Information files, but we would recommend depositing data directly to a data repository if possible.

Please update your Data Availability statement in the submission form accordingly.

5. If the reviewer comments include a recommendation to cite specific previously published works, please review and evaluate these publications to determine whether they are relevant and should be cited. There is no requirement to cite these works unless the editor has indicated otherwise. 

**Additional Editor Comments:**

This manuscript addresses an important public health issue using a valuable five-wave longitudinal dataset from Japan. The study is methodologically transparent and well aligned with the aims and scope of PLOS ONE. However, clarification and further discussion are necessary in some parts of the manuscript. Therefore, I recommend major revision.

- Page 16: "loneliness continue to represent an important public health issue", should read: "loneliness continues to represent an important public health issue."

- Lines: 124-125: “This approach is consistent with previous large-scale survey studies using the UCLA-3 scale”. Please cite the studies supporting this statement. This is particularly important because the threshold adopted produced loneliness prevalences ranging from 64% to 72%, which are considerably higher than estimates reported in most international studies.

- Please expand the discussion section. It would benefit from comparisons with findings from other longitudinal cohorts investigating loneliness trajectories during and after the COVID-19 pandemic.

- The manuscript alternates between: "COVID-19 timeline", "pandemic era" and "post-pandemic era". Consider adopting consistent terminology throughout.

- Although the authors acknowledge attrition as a limitation and provide descriptive comparisons before and after exclusion of missing data, additional discussion regarding potential selection bias is important. Individuals who remain in long-term panels may differ systematically from those lost to follow-up. They are often healthier, wealthier, and socially more engaged than those lost to follow-up, potentially influencing estimates of loneliness trajectories. The authors should discuss this issue in greater detail and, if possible, provide comparisons between retained and non-retained participants.

Reviewers' comments:

**Comments to the Author**

1. Is the manuscript technically sound, and do the data support the conclusions?

Reviewer #1: No

**********

2. Has the statistical analysis been performed appropriately and rigorously?

Reviewer #1: No

**********

3. Have the authors made all data underlying the findings in their manuscript fully available?

The PLOS Data policy

Reviewer #1: Yes

**********

4. Is the manuscript presented in an intelligible fashion and written in standard English?

Reviewer #1: Yes

**********

**Reviewer #1:**  The authors describe annual prevalence and 5-year trajectories of loneliness in a Japanese population over 5 years (2020-24). Using the UCLA loneliness scale they found that feeling lonely was a stable dimension, either as feeling lonely or never feeling lonely.

English is in my opinion good all over, Structure is logical, but there are some crucial parts that need further elaboration before this paper is fit for publishing.

Abstract – I have no comments. The authors are sufficiently cautious, and over-all abstract is consistent with the main paper.

Major revisions:

• Description of context and how the survey was conducted is limited.

• The authors do not report how many possible responders that were contacted (only how many that participated), thus they do not report the response rate. This should be done or explain why it isn’t necessary. Problem here is lack of information on representativity. I don't think the argument "the quality and representativeness of each wave were preserved through careful survey design" is sufficient to handle both age and sex skewness (see below)

• The authors do not discuss the reasons for the substantial decline of responders from the first to the second-fifth wave. The arguments presented in L95-100 could better be placed in the discussion section where limitations are discussed. If I understand correctly, only 1673 responders participated in all waves, and thus were included in the analysis, while one would expect 1/5 of 34074, approximately 6800 reponders

• The authors do not discuss age as a limitation (for participating in an online survey) and how age could be a bias in the analysis.

o From Medium.com: Only 54.2% of Japanese aged 65–74 use smartphones, compared to a staggering 85% of American seniors. A mere 7.2% of Japanese over 80 use the internet, while in the US, 44% of those 80 and older are online. The Silent Crisis: Japan’s Elderly Left Behind in the Digital Revolution | by Japan Unveiled / Akinobu Ishihara | Medium

o Could this possibly influence your results? For instance you state that ”A higher proportion of individuals under age 65 were observed in the consistently lonely group (79.8%, n=602) compared to those aged 65 or older (20.2%, n=152; χ² = 53.93, p < 0.01). One may ask – what characterizes the older participants in the data set. Do they substantially differ from the typical Japanese older adult, given that they are online users?

• The authors do not discuss the skewed sex distribution. To my understanding, 71% of the responders were males, whereas, typically, in online surveys, one would expect females to be slightly overrepresented, or at least equally represented in the data set. The substantially higher number of male responders, does, in my opinion, threaten the representativity model that the authors aimed for, as described in L95-100. Thus, I think the authors explanation on potential attrition bias (L99) is insufficient, leaving out the sex problem in the data set. This should also be discussed in the Discussion section.

Minor revisions:

• L61-63 Arguments for the particular relevance of examining loneliness in Japan, should not be “concerns about social isolation, demographic aging, and changing household structures” as this applies to many countries. However, arguing for annual mapping of loneliness in the population might be an argument, as this is probably unique for only a few countries.

• L127 This information (and reference) is already presented (in L117) and could be omitted.

• L188-190: “Specifically, while 68.5% of respondents were classified as being lonely in 2020 (the pre-pandemic era), this figure increased to 72.2% in 2021 before slightly declining to 64.3% in 2022 (during the pandemic) and reaching 65.2% in 2023 (the year the pandemic ended)”.

o This sentence may be somewhat misleading – the use of the term “slightly” might give the impression that the change from 72.2% to 64.3 percent is lesser than the change from 68.5 to 72.2% which it obviously isn’t. Consider omitting “slightly”

• L195 Table 2: Consider presenting Mean as percentages, and you should make a choice whether results should be presented in a table or in text, not in both, as here

• L214-216: “Interestingly, a noticeable proportion of participants (3.2%) felt lonely since COVID-19 and remained so after the pandemic, which is higher than the proportion of those who experienced loneliness during the COVID-19 period but recovered afterwards (2.6%)”

o This may be of interest, if the difference is significant, but you don’t report that. I think you should, and if it is not significantly different, you shouldn’t report it.

• As each variable is dichotomized, is min-max in Table 2 necessary? This also applies for table 4.

• L244 Table 4: Consider presenting Means in percentage. Age does not need 3 decimal places after the comma!

• Table 4 consider presenting median for age, in case of skewness in age distribution.

**********

what does this mean?). If published, this will include your full peer review and any attached files.

If you choose “no”, your identity will remain anonymous but your review may still be made public.

**Do you want your identity to be public for this peer review?**  For information about this choice, including consent withdrawal, please see our Privacy Policy

Reviewer #1: **Yes:**  Eivind Aakhus

**********

[NOTE: If reviewer comments were submitted as an attachment file, they will be attached to this email and accessible via the submission site. Please log into your account, locate the manuscript record, and check for the action link "View Attachments". If this link does not appear, there are no attachment files.]

To ensure your figures meet our technical requirements, please review our figure guidelines: https://journals.plos.org/plosone/s/figures

You may also use PLOS’s free figure tool, NAAS, to help you prepare publication quality figures: https://journals.plos.org/plosone/s/figures#loc-tools-for-figure-preparation.

NAAS will assess whether your figures meet our technical requirements by comparing each figure against our figure specifications.

# Author comment: Author response to Decision Letter 1

**DOI:** 10.1371/journal.pone.0358269.r002

25 Jul 2026

Response to Reviewers

Manuscript ID: PONE-D-26-09247

Title: The Dynamics of Loneliness: A Longitudinal Exploration of Transitions in Japan Across the COVID-19 Period, 2020–2024

Dear Academic Editor and Reviewer 1,

Thank you very much for your careful and constructive evaluation of our manuscript. We have revised the manuscript substantially in response to the comments.

The principal revisions include a more detailed description of the survey administration and participant flow; clarification of the baseline recruitment procedure and follow-up response counts; a new comparison between respondents included and not included in the final analytic sample; expanded discussion of attrition, selection bias, online-panel participation, age-related digital access, and the male-skewed sample; clarification of the UCLA-3 threshold; a sensitivity analysis using a stricter definition of loneliness; expanded comparison with other longitudinal studies; and revised presentation of the descriptive tables.

We have also removed the previous claims concerning representativeness and now interpret the results as descriptive findings among respondents retained in the five-wave online panel.

Our point-by-point responses are provided below.

Responses to the Academic Editor

Comment 1

Page 16: “loneliness continue to represent an important public health issue,” should read: “loneliness continues to represent an important public health issue.”

Response:

Thank you. During revision, we condensed this part of the Results section and removed the sentence containing the grammatical error. The Results now provide a concise description of the annual pattern.

________________________________________

Comment 2

Lines 124–125: “This approach is consistent with previous large-scale survey studies using the UCLA-3 scale.” Please cite the studies supporting this statement. This is particularly important because the threshold adopted produced loneliness prevalences ranging from 64% to 72%, which are considerably higher than estimates reported in most international studies.

Response:

We agree that the operational definition required clearer explanation and support. We have added citations to relevant previous large-scale Japanese online-panel studies using the UCLA-3 items and the same inclusive coding approach.

We now explain explicitly that the primary definition classifies respondents as lonely when they answer “some of the time” or “often” to at least one of the three UCLA items. We acknowledge that this inclusive threshold produces relatively high prevalence estimates.

We also added a sensitivity analysis using a stricter definition, under which respondents were classified as lonely only if they answered “often” to at least one item. The stricter definition substantially reduced annual prevalence estimates. However, prevalence continued to peak in 2021, and stable trajectories remained more common than transitional trajectories. We also clarify that the distribution and ranking of specific five-year sequences changed under the stricter definition.

These revisions appear in Sections 2.2, 2.3, and 3.4, the Discussion, and Appendix B.

________________________________________

Comment 3

Please expand the discussion section. It would benefit from comparisons with findings from other longitudinal cohorts investigating loneliness trajectories during and after the COVID-19 pandemic.

Response:

We have expanded the Discussion to compare our findings with repeated nationwide survey evidence from the JACSIS 2020–2021 study, a two-year longitudinal study conducted in Japan, and longitudinal evidence from the Swiss Household Panel.

The revised Discussion explains that previous studies generally identified worsening loneliness during the earlier phase of the pandemic, while subsequent patterns varied across populations. We also clarify that the present study extends this literature by examining five annual waves from 2020 to 2024.

________________________________________

Comment 4

The manuscript alternates between “COVID-19 timeline,” “pandemic era,” and “post-pandemic era.” Consider adopting consistent terminology throughout.

Response:

We agree. We revised the terminology throughout the body of the manuscript to use more precise descriptions of the study periods. These now include the February 2020 baseline, the pandemic-period waves, and the 2024 follow-up conducted after the WHO determined that COVID-19 no longer constituted a Public Health Emergency of International Concern.

We also removed the previous wording suggesting that the pandemic itself “ended” in 2023.

________________________________________

Comment 5

Although the authors acknowledge attrition as a limitation and provide descriptive comparisons before and after exclusion of missing data, additional discussion regarding potential selection bias is important. Individuals who remain in long-term panels may differ systematically from those lost to follow-up. They are often healthier, wealthier, and socially more engaged than those lost to follow-up, potentially influencing estimates of loneliness trajectories. The authors should discuss this issue in greater detail and, if possible, provide comparisons between retained and non-retained participants.

Response:

We agree and have made several substantial revisions.

First, we added a comparison of baseline characteristics between respondents included and not included in the final analytic sample. The results are presented in Appendix A and include standardized mean differences.

The comparison showed differences in several observed characteristics. Respondents included in the final analytic sample were older, more likely to be male and university educated, and had higher financial literacy and household assets.

We therefore expanded the Discussion to acknowledge potential selection bias and the possibility of additional unobserved differences. The revised manuscript explicitly states that the findings should be interpreted as descriptive patterns among retained online-panel respondents rather than as nationally representative prevalence estimates.

________________________________________

Responses to Reviewer 1

We thank Reviewer 1 for the careful review and constructive comments.

General comment on the Abstract

Abstract – I have no comments. The authors are sufficiently cautious, and the overall abstract is consistent with the main paper.

Response:

Thank you for this positive assessment. Although no specific revision was required in response to this comment, we updated the Abstract to ensure consistency with the revised Methods, sensitivity analysis, and more cautious interpretation of generalizability.

________________________________________

Major comments

Comment 1

Description of context and how the survey was conducted is limited.

Response:

We agree. Section 2.1 has been substantially expanded.

The revised manuscript now identifies Nikkei Research as the survey administrator; describes the use of its own and allied registered online monitor panels; provides the survey dates for all five waves; explains the baseline target-sample recruitment procedure; and reports the numbers of distributed invitations and effective completed responses for the follow-up waves.

We also clarify that the participants were adults aged 20 years or older residing throughout Japan.

________________________________________

Comment 2

The authors do not report how many possible responders were contacted and thus do not report the response rate. This should be done or explain why it is not necessary. The argument that the quality and representativeness of each wave were preserved is insufficient.

Response:

Thank you. We have removed the previous statement that the quality and representativeness of each wave were preserved.

For the 2020 baseline survey, the authors requested approximately 17,000 completed responses, and the survey company distributed invitations until the target sample size was reached. The total number of invitations required to reach the target was not available to the authors. Consequently, the baseline response rate could not be calculated. This is now stated explicitly in both the Data section and the limitations.

For the follow-up waves, we now report the numbers of distributed invitations and effective completed responses:

• 2021: 16,949 invitations and 6,103 effective responses

• 2022: 6,103 invitations and 4,281 effective responses

• 2023: 4,221 invitations and 3,410 effective responses

• 2024: 3,486 invitations and 2,929 effective responses

We no longer interpret the analytic sample as nationally representative.

________________________________________

Comment 3

The authors do not discuss the reasons for the substantial decline of responders from the first to the second-fifth wave. If I understand correctly, only 1,673 responders participated in all waves and were included in the analysis, while one would expect approximately 6,800 respondents.

Response:

We agree that the participant flow required clearer explanation.

The revised Data section now distinguishes among the effective responses collected at each wave, the matched five-wave sample, and the final complete-case analytic sample. The 2024 survey yielded 2,929 effective completed responses. After matching respondents across all five waves, 2,817 individuals remained. After excluding observations with missing information on variables required for the analysis, the final analytic sample consisted of 1,673 respondents.

We also explain that some respondents could not be invited to later waves because they had withdrawn from the survey company’s monitor panel. Noncompletion among those invited and complete-case exclusion also contributed to the reduction in sample size.

The sum of the wave-specific sample counts does not represent the number of distinct individuals, because respondents could participate in multiple waves. The final analysis required the same individual to be matched across all five waves and to have complete information on the variables used.

The implications of the resulting attrition and complete-case restriction are now discussed explicitly, and Appendix A compares respondents included and not included in the final analytic sample.

________________________________________

Comment 4

The authors do not discuss age as a limitation for participating in an online survey and how age could be a source of bias. What characterizes the older participants in the dataset? Do they differ from typical Japanese older adults, given that they are online users?

Response:

We agree that continued participation in an online panel may select a particular group of older adults.

We added a specific limitation explaining that older adults who repeatedly participate in online surveys may differ from older adults with limited internet access or lower digital familiarity. They may also differ in their health, socioeconomic resources, and capacity to participate in repeated surveys.

The present dataset does not permit a direct comparison between the older participants and the entire older population of Japan. We therefore do not claim that the older respondents are representative of all older Japanese adults. Instead, the revised manuscript states that the age-related patterns may not generalize to the broader older population.

________________________________________

Comment 5

The authors do not discuss the skewed sex distribution. Approximately 71% of the respondents were male, which threatens the representativeness argument. This should be discussed in the Discussion section.

Response:

We agree. We removed the previous claims concerning representativeness and added a separate limitation addressing the male-skewed analytic sample.

The revised manuscript states that men accounted for 71.1% of the final analytic sample and acknowledges that this imbalance may have influenced both the prevalence estimates and trajectory distributions. We therefore interpret the results as descriptive findings among retained panel respondents rather than as nationally representative estimates.

________________________________________

Minor comments

Comment 6

Lines 61–63: The particular relevance of examining loneliness in Japan should not be based on social isolation, demographic aging, and changing household structures, because these apply to many countries. Annual mapping may be a more relevant argument.

Response:

We agree. We replaced the previous justification with a more specific rationale.

The revised Introduction now explains that Japan provides a useful setting because the same nationwide online survey was conducted annually over five consecutive years, enabling assessment of both annual prevalence and individual-level stability and transition patterns.

________________________________________

Comment 7

Line 127: This information and reference are already presented and could be omitted.

Response:

We removed the redundant description and consolidated the explanation of the UCLA-3 measure, its coding, and its internal consistency in Section 2.2.

Comment 8

Lines 188–190: The word “slightly” may be misleading when describing the decline from 72.2% to 64.3%.

Response:

We agree. The word “slightly” and the original sentence were removed. The Results now state concisely that loneliness prevalence peaked in 2021 and was lower and relatively stable from 2022 to 2024.

________________________________________

Comment 9

Table 2: Consider presenting mean values as percentages, and results should not be repeated in both the table and the text.

Response:

Table 2 has been reformatted to present the number and percentage of respondents classified as lonely in each year.

We also shortened the accompanying text so that it summarizes only the principal pattern instead of repeating every value reported in the table.

________________________________________

Comment 10

The comparison between 3.2% who remained lonely and 2.6% who recovered should be supported by a significance test; otherwise, it should not be reported.

Response:

We agree. The comparison and the word “Interestingly” were removed. We now describe the less frequent trajectories without claiming that the difference between 3.2% and 2.6% is statistically meaningful.

________________________________________

Comment 11

As each variable is dichotomized, is min-max in Table 2 necessary? This also applies to Table 4.

Response:

We agree. Minimum and maximum values have been removed for dichotomous variables.

Table 2 now reports counts and percentages. Table 4 reports categorical variables as n (%) and continuous variables using appropriate descriptive statistics.

________________________________________

Comment 12

Table 4: Consider presenting means as percentages. Age does not need three decimal places.

Response:

Table 4 has been revised accordingly. Categorical variables are now presented as n (%), and age is no longer reported to three decimal places.

________________________________________

Comment 13

Table 4: Consider presenting median age in case the age distribution is skewed.

Response:

We agree. Table 4 now reports the median age and interquartile range. The median age in 2024 was 57 years, with an interquartile range of 49–66 years.

We again thank the Academic Editor and Reviewer 1 for their thoughtful comments. We believe that the revisions have improved the transparency, precision, and interpretation of the manuscript.

Sincerely,

Yoshihiko Kadoya

Corresponding Author

On behalf of all authors

Attachment Submitted filename: Response to Reviewers.docx

# Peer review: Decision Letter 1

**DOI:** 10.1371/journal.pone.0358269.r003

**Authors:** Bruna Luchesi

30 Aug 2026

The Dynamics of Loneliness: A Longitudinal Exploration of Transitions in Japan Across the COVID-19 Period, 2020–2024

PONE-D-26-09247R1

Dear Dr. Kadoya,

We’re pleased to inform you that your manuscript has been judged scientifically suitable for publication and will be formally accepted for publication once it meets all outstanding technical requirements.

Within one week, you’ll receive an e-mail detailing the required amendments. When these have been addressed, you’ll receive a formal acceptance letter and your manuscript will be scheduled for publication.

An invoice will be generated when your article is formally accepted. Please note, if your institution has a publishing partnership with PLOS and your article meets the relevant criteria, all or part of your publication costs will be covered. Please make sure your user information is up-to-date by logging into Editorial Manager at Editorial Manager® and clicking the ‘Update My Information' link at the top of the page. For questions related to billing, please contact billing support.

If your institution or institutions have a press office, please notify them about your upcoming paper to help maximize its impact. If they’ll be preparing press materials, please inform our press team as soon as possible -- no later than 48 hours after receiving the formal acceptance. Your manuscript will remain under strict press embargo until 2 pm Eastern Time on the date of publication. For more information, please contact onepress@plos.org.

Kind regards,

Bruna Moretti Luchesi, Ph.D.

Academic Editor

PLOS One

Additional Editor Comments (optional):

The revisions satisfactorily address the concerns raised in the previous round, and the manuscript is now clearer and more consistent. No further major issues were identified during the current evaluation.

I therefore recommend acceptance of the manuscript for publication in its current form.

Reviewers' comments:

Reviewer's Responses to Questions

**Comments to the Author**

Reviewer #1: All comments have been addressed

**********

2. Is the manuscript technically sound, and do the data support the conclusions?

Reviewer #1: Yes

**********

3. Has the statistical analysis been performed appropriately and rigorously?

Reviewer #1: N/A

**********

4. Have the authors made all data underlying the findings in their manuscript fully available?

The PLOS Data policy

Reviewer #1: Yes

**********

5. Is the manuscript presented in an intelligible fashion and written in standard English?

Reviewer #1: Yes

**********

Reviewer #1: I think all my concerns have been addressed. The article appears better, more readable and better structured.

**********

what does this mean?). If published, this will include your full peer review and any attached files.

If you choose “no”, your identity will remain anonymous but your review may still be made public.

**Do you want your identity to be public for this peer review?**  For information about this choice, including consent withdrawal, please see our Privacy Policy

Reviewer #1: No

**********

# Editor report: Acceptance letter

**DOI:** 10.1371/journal.pone.0358269.r004

**Authors:** Bruna Luchesi

PONE-D-26-09247R1

PLOS One

Dear Dr. Kadoya,

I'm pleased to inform you that your manuscript has been deemed suitable for publication in PLOS One. Congratulations! Your manuscript is now being handed over to our production team.

At this stage, our production department will prepare your paper for publication. This includes ensuring the following:

* All references, tables, and figures are properly cited

* All relevant supporting information is included in the manuscript submission,

* There are no issues that prevent the paper from being properly typeset

You will receive further instructions from the production team, including instructions on how to review your proof when it is ready. Please keep in mind that we are working through a large volume of accepted articles, so please give us a few days to review your paper and let you know the next and final steps.

Lastly, if your institution or institutions have a press office, please let them know about your upcoming paper now to help maximize its impact. If they'll be preparing press materials, please inform our press team within the next 48 hours. Your manuscript will remain under strict press embargo until 2 pm Eastern Time on the date of publication. For more information, please contact onepress@plos.org.

You will receive an invoice from PLOS for your publication fee after your manuscript has reached the completed accept phase. If you receive an email requesting payment before acceptance or for any other service, this may be a phishing scheme. Learn how to identify phishing emails and protect your accounts at https://explore.plos.org/phishing.

If we can help with anything else, please email us at customercare@plos.org.

Thank you for submitting your work to PLOS One and supporting open access.

Kind regards,

PLOS One Editorial Office Staff

on behalf of

Dr. Bruna Moretti Luchesi

Academic Editor

PLOS One
